# Atrial lead entanglement during transcatheter mitral valve-in-ring implantation in patient with dual-chamber implantable cardioverter-defibrillator: case report

**DOI:** 10.1093/ehjcr/ytaf653

**Published:** 2026-01-05

**Authors:** Suryo Ardi Hutomo, Xiang Chen, Bin Wang, Yan Er Yao, Yan Wang

**Affiliations:** Department of Cardiology, Xiamen Cardiovascular Hospital Xiamen University, 2999 Jinshan Road, Huli District, Xiamen City, Fujian Province 361004, China; Cardiovascular Subspecialist Study Program, Department of Cardiology and Vascular Medicine, Faculty of Medicine, Universitas Airlangga – Dr. Soetomo General Academic Hospital, Jl. Mayjen. Prof. Dr. Moestopo 47, Surabaya 60131, Indonesia; Cardiovascular Department, Surabaya Central General Hospital, Jl. Indrapura No.17 Surabaya 60176, Indonesia; Department of Cardiology, Xiamen Cardiovascular Hospital Xiamen University, 2999 Jinshan Road, Huli District, Xiamen City, Fujian Province 361004, China; Department of Cardiology, Xiamen Cardiovascular Hospital Xiamen University, 2999 Jinshan Road, Huli District, Xiamen City, Fujian Province 361004, China; Department of Cardiology, Xiamen Cardiovascular Hospital Xiamen University, 2999 Jinshan Road, Huli District, Xiamen City, Fujian Province 361004, China; Department of Cardiology, Xiamen Cardiovascular Hospital Xiamen University, 2999 Jinshan Road, Huli District, Xiamen City, Fujian Province 361004, China

**Keywords:** Mitral valve intervention, Ring annuloplasty failure, Valve-in-ring, Dual-chamber ICD, Atrial lead, Case report

## Abstract

**Background:**

Ring annuloplasty failure after mitral valve repair frequently occurs after long-term follow up. Valve-in-ring transcatheter mitral valve replacement (TMVR) is an emerging therapeutic option for this high surgical risk condition. While a dual-chamber implantable cardioverter-defibrillator (ICD) is frequently utilized for sudden cardiac death prevention in this population, valve-in-ring TMVR intraprocedural difficulty associated with atrial lead presence is rarely known.

**Case summary:**

A 76-year-old male, with history of coronary artery bypass graft surgery and mitral ring annuloplasty experienced recurrence of severe mitral regurgitation. Due to high surgical mortality risk and unsuitable anatomy of mitral valve for transcatheter edge-to-edge repair, this patient underwent mitral valve-in-ring TMVR. Using transvenous-transseptal access, a 26 mm SAPIEN-3 prosthetic valve was delivered to the mitral area. However, as the patient has a history of dual-chamber ICD, a prosthetic valve cannot pass through during delivery, due to atrial lead entanglement. To overcome this difficulty, atrial septum balloon dilatation manoeuvre was done to facilitate valve delivery, which resulted in successful prosthetic valve delivery and deployment at the mitral position with satisfactory results.

**Discussion:**

Valve delivery difficulty due to atrial lead entanglement can occur during valve-in-ring TMVR in patients with dual-chamber ICD implantation history. Atrial septum balloon dilatation can be performed to overcome this problem.

Learning points

- During valve-in-ring transcatheter mitral valve replacement for mitral ring annuloplasty failure in patients with a history of dual-chamber implantable cardioverter-defibrillator (ICD) implantation, an operator may experience difficulty, in the form of atrial lead entanglement during transcatheter prosthetic valve delivery.
- Atrial septal balloon dilatation can be used as a bailout in overcoming atrial lead entanglement, to facilitate valve-in-ring procedures in patients with dual-chamber ICD.

## Introduction

Mitral valve (MV) repair with ring annuloplasty is a recommended procedure for severe functional mitral regurgitation (MR) associated with ischaemic heart failure (HF), in patients undergoing surgical coronary revascularization.^1^ However, the incidence of ring annuloplasty failure, which presents as severe MR recurrence, remains high—particularly after long-term follow-up—and often necessitates re-intervention.^2,3^ Surgical reoperation has high mortality risk,^4^ bringing the role of transcatheter percutaneous valve-in-ring intervention using a transcatheter aortic valve replacement device,^5^ which is less invasive and feasible and has better outcomes.^6^

Patients with ischaemic HF and reduced ejection fraction (EF) are at high risk of sudden cardiac death (SCD) from malignant arrhythmias, necessitating the implantation of an implantable cardioverter-defibrillator (ICD) for primary prevention.^7^ Even without a pacing indication, dual-chamber ICD (DD-ICD) is often implanted with an added atrial lead to reduce inappropriate shocks.^8^ This case highlights the challenges of performing a valve-in-ring transcatheter mitral valve replacement (TMVR) due to the presence of the ICD atrial lead.

## Summary figure

**Figure:**
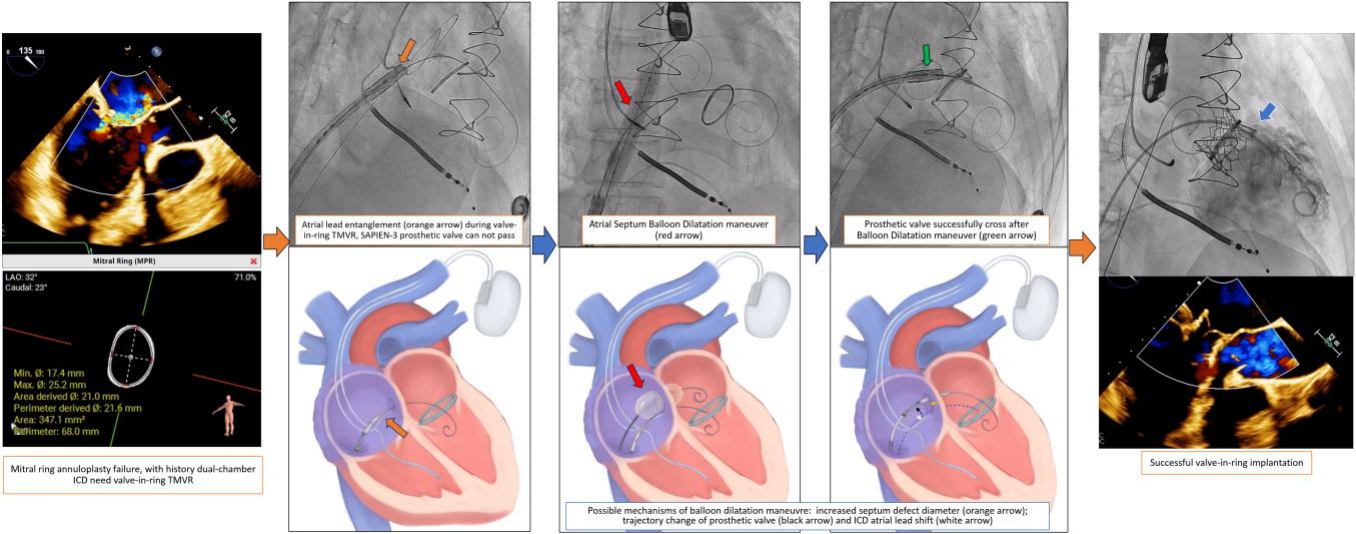


## Case presentation

A 76-year-old man presented with worsening shortness of breath over 10 days and history of recurrent hospitalizations for HF and arrhythmias. He had a history of coronary artery bypass graft surgery and mitral annuloplasty using a 26 mm Physio-I ring (Edwards Lifesciences, Irvine, California) 12 years ago, for indication of three-vessel coronary artery disease, ischaemic HF, and severe functional MR. He also had catheter ablation 7 years ago for recurrent atrial and ventricular tachycardia and had DD-ICD implanted for the primary prevention of SCD.

**Figure 1:**
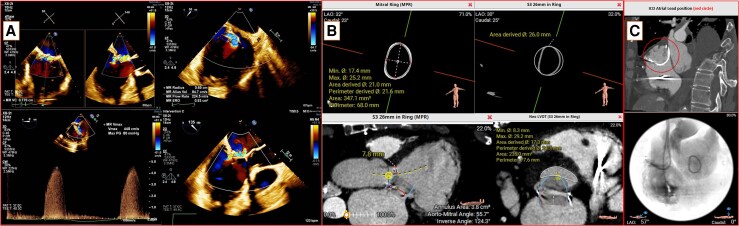
(*A*) Echocardiography assessment showed the recurrence of severe mitral regurgitation. (*B*) CT analysis before valve-in-ring transcatheter mitral valve replacement. (*C*) CT evaluation of implantable cardioverter-defibrillator atrial lead position.

Transthoracic echocardiography showed left atrial (LA) and left ventricular (LV) dilatation, decreased LV systolic function (31% EF), reduced posterior wall motion, and moderate-to-severe MR. Transoesophageal echocardiography (TEE) showed complete mitral ring, anterior and posterior mitral leaflet restricted motion, causing “severe MR” [77 mm vena contracta, effective regurgitant orifice (ERO) of 0.53 cm^2^] and reduced MV area of ​​3.19 cm^2^ (*Figure 1A*).

Multidisciplinary team discussion was conducted to determine the best management for this patient. With the presence of severe MR recurrence accompanied by repeated episodes of arrhythmia and HF hospitalization, this patient has strong indication for MV re-intervention. Given high risk of open heart surgery (EuroSCORE, 7), then transcatheter percutaneous intervention is considered the best choice. Valve-in-ring TMVR was planned to be performed using SAPIEN-3 valve (Edwards Lifesciences, Irvine, California). Computed tomography (CT) scan analysis (*Figure 1B* and *C*), performed before valve-in-ring TMVR, showed complete mitral annular ring, with ​​347.1 mm^2^ internal area. Simulation using a 26 mm SAPIEN-3 valve showed a neo-left ventricular outflow tract (LVOT) area of ​​2.35 cm^2^, suggesting low risk of post-procedural LVOT obstruction.^9^ Atrial ICD leads also appreciated lie from posterior to anterior.

Valve-in-ring TMVR was conducted under general anaesthesia, with the transvenous-transseptal approach. A temporary pacing electrode was inserted from the left femoral vein. Under guidance of TEE, the atrial septum was punctured, and an 8.5F Agilis (Abbott, USA) steerable catheter was introduced to the LA. The measured preoperative pressure was 26/18 mmHg. Then, the long Angelguide® (MicroPort, Shanghai, China) stiff-wire was directed to pass from the MV to LV. Left ventricular angiography was performed and Grade III MR is appreciated.

**Figure 2:**
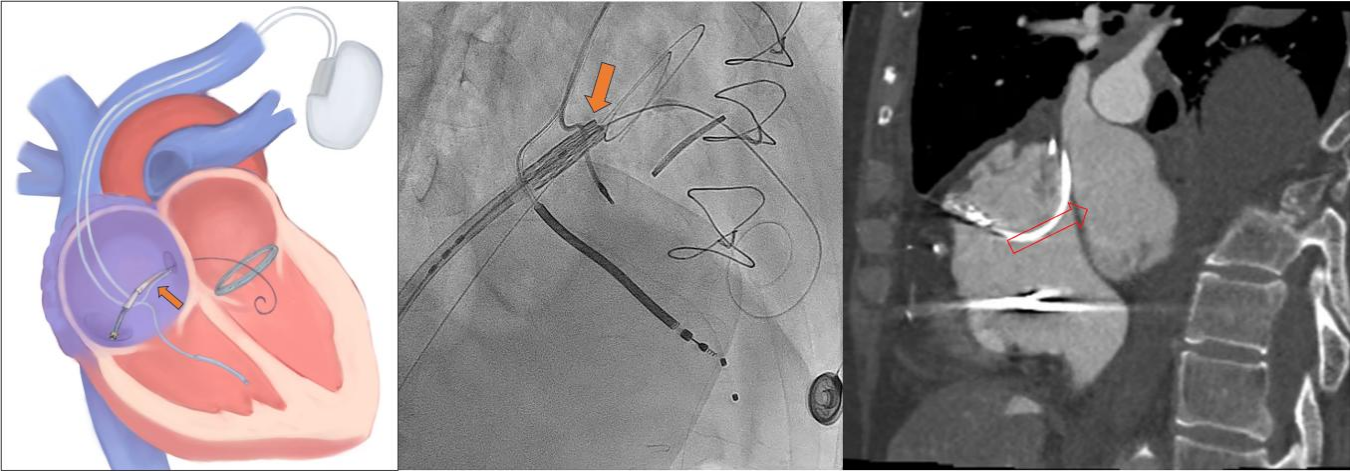
Schematic diagram, fluoroscopy, and CT images* showing the failed advancement of a SAPIEN-3 prosthetic valve due to entanglement with an implantable cardioverter-defibrillator atrial lead (*transparent arrow represents the SAPIEN-3 prosthetic valve).

After securing access to the LA with 0.018′ wire and to the LV with an Angelguide® wire, a 14F E-sheath (Edwards Lifesciences, Irvine, California) was inserted. Next, a 12 × 40 mm Armada balloon was advanced along the stiff-wire to repeatedly dilate the atrial septum. Following septal dilation, a 26 mm SAPIEN-3 valve was prepared for delivery to the mitral area via the right atrium (RA). However, advancement of the valve was unsuccessful due to entanglement with the ICD atrial lead. Several additional attempts to pass the valve through the atrial lead were made, but all were unsuccessful (*Figure 2*).

**Figure 3:**
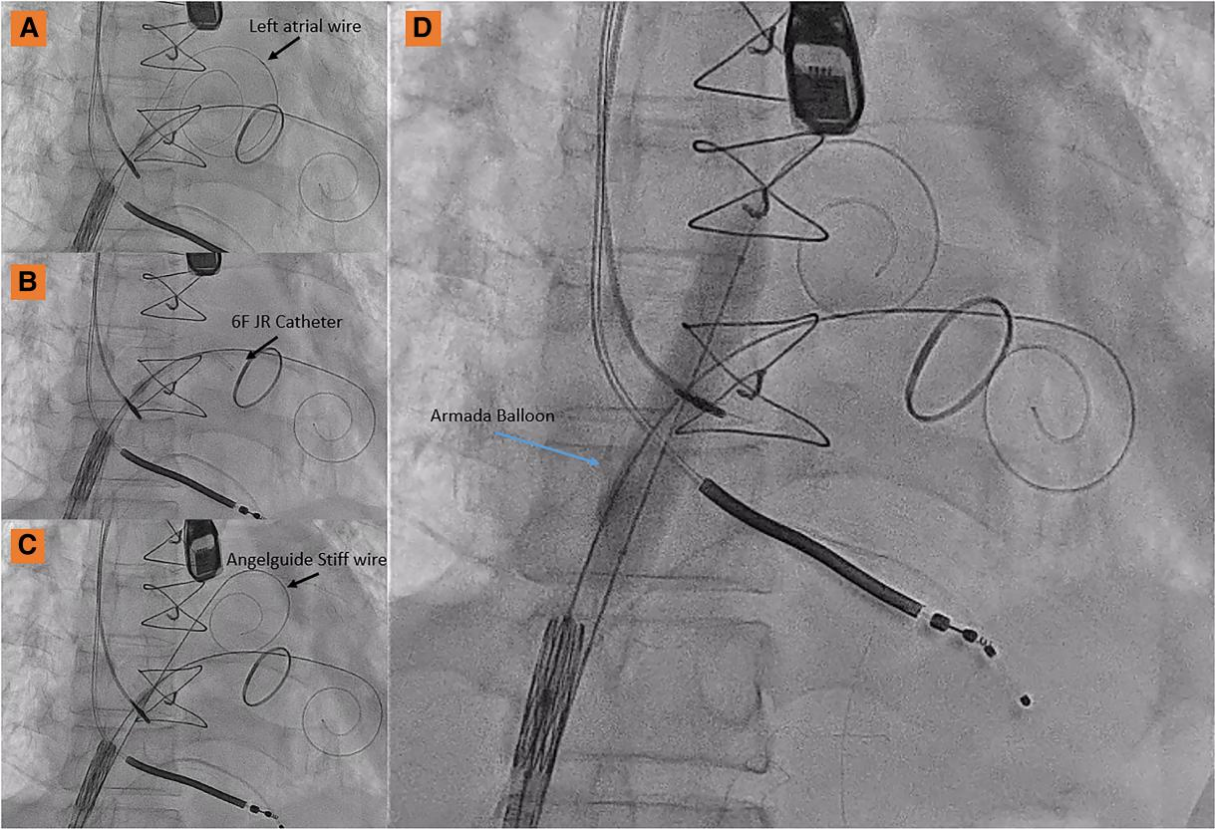
Atrial septum balloon dilatation manoeuvre.

To overcome this difficulty, the following sequence was performed to achieve atrial septum balloon dilatation: first, a 6F arterial sheath was inserted along the LA wire. Next, the LA wire was exchanged for an Angelguide® wire using JR catheter guidance (*Figure 3A–C*). Finally, 5 × 80 mm and then 14 × 60 mm Armada balloons were sequentially advanced along the LA Angelguide® wire, dilating the atrial septum several times (*Figure 3D*).

**Figure 4:**
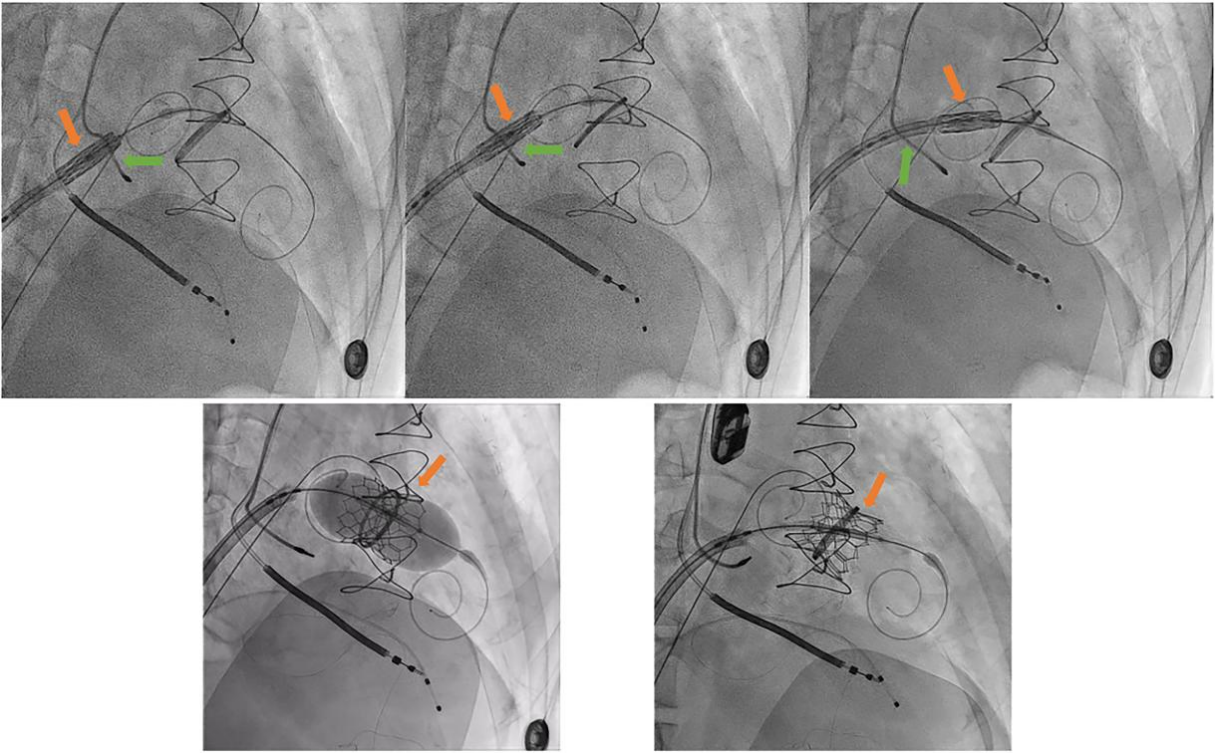
The prosthetic valve can pass through the atrial lead after balloon dilatation and successfully deployed in the mitral position.

After balloon dilatation was done and withdrawn, a 26 mm SAPIEN-3 valve was reintroduced and successfully advanced through the atrial lead, to the septum and finally to the mitral position. Under 160 b.p.m. pacing rate, the valve was successfully inflated with a 23 mL balloon and post-dilated with additional 2 mL. The valve delivery system was withdrawn without difficulty after successful valve implantation (*Figure 4*).

**Figure 5:**
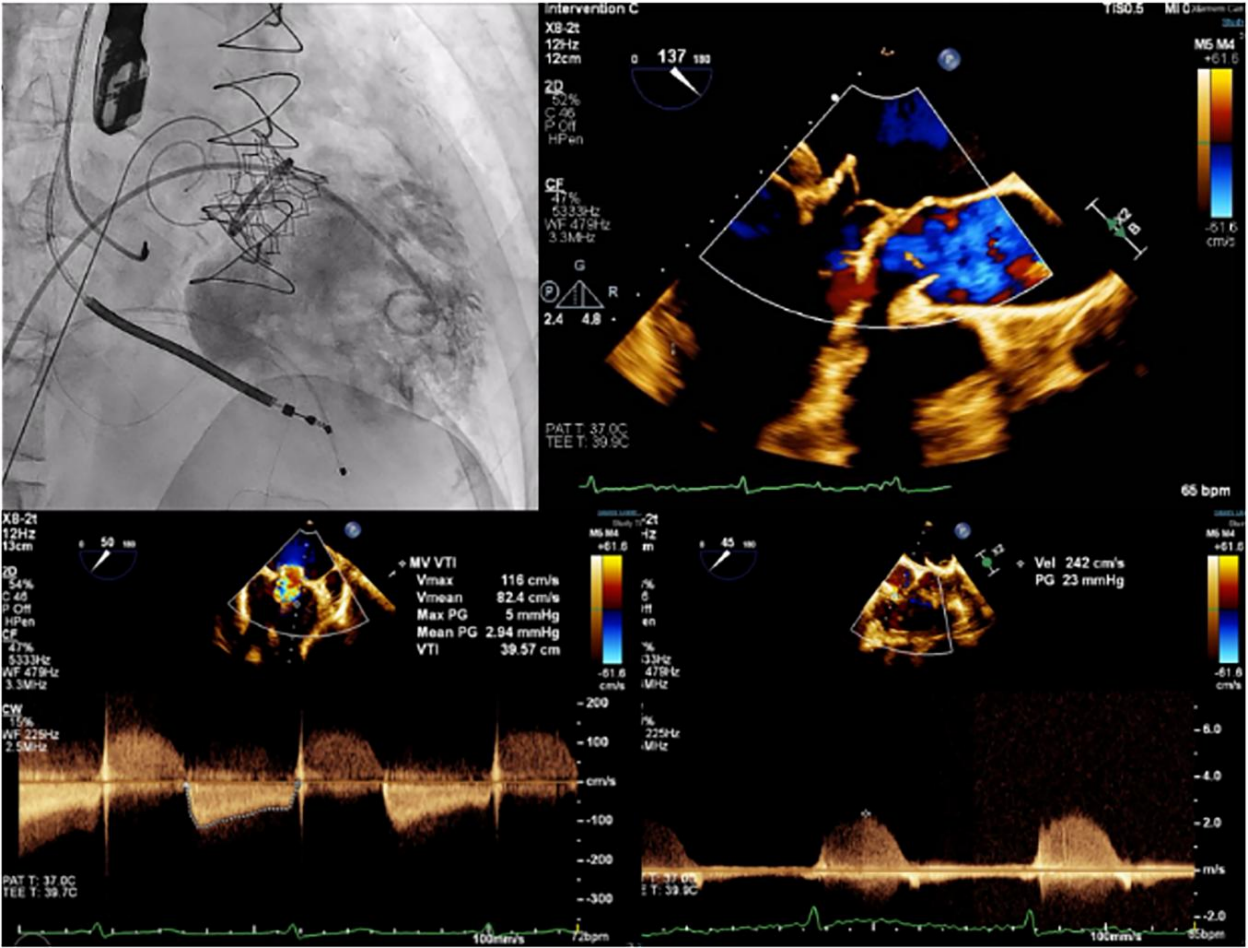
Satisfactory result after mitral valve-in-ring implantation showed no residual mitral regurgitation, no paravalvular leakage, and low *trans*-mitral pressure gradient.

Following the valve-in-ring implantation, LV angiography showed no MR. The *trans*-mitral pressure gradient was 2 mmHg, and the LA pressure decreased to 15/9 mmHg. Echocardiography confirmed good valve function with no paravalvular leakage or pericardial effusion. The mean pressure gradient across the MV was minimal at 2.54 mmHg, with a slight left-to-right shunt (*Figure 5*). The patient remained stable during post-procedure observation and was discharged after 3 days. An evaluation of the ICD showed normal atrial and ventricular lead pacing and sensing functions.

## Discussion

This case involves a failed mitral ring annuloplasty in a patient with ischaemic cardiomyopathy and recurrent HF hospitalization. Severe MR can be treated with valve-in-ring TMVR, as complications from the ICD atrial lead have been addressed. Given the high surgical risk, valve-in-ring TMVR is the preferred option, while transcatheter edge-to-edge repair (TEER) was not chosen due to its complexity and risk of mitral stenosis.^10^

Transcatheter mitral valve replacement is an emerging treatment option for MV repair surgery failure.^5,6^ However, TMVR in ring annuloplasty is more challenging than in degenerated bioprosthetic valves. Registries indicate that valve-in-ring TMVR often yields worse outcomes and lower technical success rates. Challenges include paravalvular leakage due to the rigid mitral ring, LVOT obstruction from an excessively long MV, and potential valve migration or embolization. In this case, we encountered a rare technical difficulty in delivering the prosthetic valve from the RA to the mitral position, primarily due to the entanglement of the ICD atrial leads.

Atrial septum balloon dilatation is a routine step in the TMVR procedure to achieve access from the RA to the LA. In this case, repeated dilatation at the atrial septum with escalating balloon sizes was performed as a critical step to facilitate prosthetic valve crossing. It functions as a slider, used to separate the valve system from the pacing lead, allowing the valve system to smoothly cross the atrial septum. This manoeuvre benefited the procedure in three ways: it increased the diameter of the septum defect to reduce resistance; it helped the wire trajectory move away from the atrial lead; and it slightly displaced the atrial lead position (Summary figure). When using this technique, it is essential to proceed carefully so as not to lose access to the LA, to assess for the presence of thrombus, to evaluate for residual septal defects, and to check post-operative atrial lead function.

If this manoeuvre is unsuccessful, it is worth considering other possible causes of difficulty in advancing the prosthetic valve other than atrial lead entanglement, such as interference from the MV ring due to non-coaxial alignment. Using the left anterior oblique (LAO) view during the procedure (which can separate the atrial septum from the atrial lead) may have helped in clarifying the underlying cause of the advancing difficulty. Lastly, if needed, a transapical approach can be considered as a last resort, weighing the associated benefits and risks, which may include myocardial or coronary injury, infection, and bleeding.^11^

## Conclusion

Valve delivery difficulty due to atrial lead can occur during valve-in-ring TMVR in patients with a history of DD-ICD implantation. Atrial septum balloon dilatation can be performed to overcome this problem.

## Lead author biography


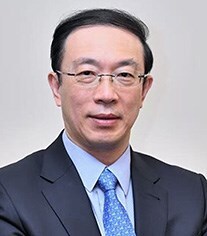


Professor Wang Yan is the President of Xiamen Cardiovascular Hospital Xiamen University. He is a chief physician (Level II), a professor at Xiamen University, and a doctoral supervisor. He has been recognized as a National Distinguished Physician and an Outstanding Young and Middle-Aged Expert by China’s National Health Commission and is a recipient of the Special Government Allowance of the State Council. He is also a fellow of several prestigious international academic organizations, including the American College of Cardiology (FACC), the European Society of Cardiology (FESC), and the Society for Cardiovascular Angiography and Interventions (FSCAI).

## Supplementary Material

ytaf653_Supplementary_Data

## Data Availability

All available data are presented within the manuscript and its online Supplementary material.
